# G.P. and Hospital Medicine

**Published:** 1988-02

**Authors:** Donald Ractliffe

**Affiliations:** 12 St Hilary Close BRISTOL BS9 1DA


### Correspondence

**General practice and hospital medicine – a new relationship?**

Sir,

Dr Michael Whitfield's contribution (Vol. 102(iv) November 1987 pp 95–6) raises some important issues.

He begins by reviewing replies from today's students to his question: "Has this attachment (to a general practitioner) altered your previous view about hospital medicine?" One student commented: "The attitude of SHOs to GPs' letters seems a touch arrogant on occasions considering they have no background knowledge of patients." Assuming today's SHOs have had the same fortnight's contact with general practice, some of them have clearly not gained a beneficial lasting impression. This could be because they found themselves in less good practices; it could be due to weightier influence of their senior colleagues; it might be that the arrogance is only an occasional outburst of exasperation under pressure; or, indeed, that some crucial "background knowledge" is missing from the referral letter. Another student realised: "How important it is for hospital staff not to make disparaging remarks about the patient's GP's skills in the presence of the patient." The GMC specifically warns us that "... such disparagement may raise a question of serious professional misconduct."

Dr Whitfield is however "bothered" by the fact that *some* students "will continue in their careers to propagate the traditional teaching hospital attitudes." I also am bothered—and disappointed—that, rather more than fifteen years since the start of Bristol's "Medicine-in-the Community" course and only slightly less time since the introduction of vocational training (with its emphasis on the integration of hospital and general practice experience), the situation is as Michael describes. One wonders how long it takes to change attitudes.

The article concludes: "We are both at fault for the difficulties and we can both strive for an improvement in the relationship." Just so, but meanwhile we might reflect that it *is* possible to be frank without being offensive; it *is* possible to be critical without being uncharitable; and it should be possible for both "sides" to listen to criticism and learn from it, rather than reacting in a paranoid fashion—which is self-defeating. But it needs tact and understanding of each other's difficulties—yes, and courage. At all events it is better to be open than superficially polite yet insincere.

To think the worst of one's colleagues is to deserve the consequences.

Yours sincerely,

Donald Ractliffe
12 St Hilary Close
BRISTOL BS9 1DA

